# Neuroprotective Effect of Kinase Inhibition in Ischemic Factor Modeling In Vitro

**DOI:** 10.3390/ijms22041885

**Published:** 2021-02-14

**Authors:** Elena V. Mitroshina, Maria M. Loginova, Maria O. Savyuk, Mikhail I. Krivonosov, Tatiana A. Mishchenko, Viktor S. Tarabykin, Mikhail V. Ivanchenko, Maria V. Vedunova

**Affiliations:** 1 Institute of Biology and Biomedicine, Lobachevsky State University of Nizhni Novgorod, 23 Prospekt Gagarina, 603950 Nizhny Novgorod, Russia; pandaagron@yandex.ru (M.M.L.); mary.savyuk@bk.ru (M.O.S.); saharnova87@mail.ru (T.A.M.); Victor.Tarabykin@charite.de (V.S.T.); 2 Institute of Information, Technology, Mathematics and Mechanics, Lobachevsky State University of Nizhni Novgorod, 23 Prospekt Gagarina, 603950 Nizhny Novgorod, Russia; mike_live@mail.ru (M.I.K.); ivanchenko.mv@gmail.com (M.V.I.); 3 Institute of Cell Biology and Neurobiology, Charité—Universitätsmedizin Berlin, Charitéplatz 1, 10117 Berlin, Germany

**Keywords:** protein kinase inhibitors, primary hippocampal cultures, functional neural network activity, neuroprotection, ischemia, hypoxia

## Abstract

The contribution of many neuronal kinases to the adaptation of nerve cells to ischemic damage and their effect on functional neural network activity has not yet been studied. The aim of this work is to study the role of the four kinases belonging to different metabolic cascades (SRC, Ikkb, eEF2K, and FLT4) in the adaptive potential of the neuron-glial network for modeling the key factors of ischemic damage. We carried out a comprehensive study on the effects of kinases blockade on the viability and network functional calcium activity of nerve cells under ischemic factor modeling in vitro. Ischemic factor modelling was performed on day 14 of culturing primary hippocampal cells obtained from mouse embryos (E18). The most significant neuroprotective effect was shown in the blockade of FLT4 kinase in the simulation of hypoxia. The studies performed revealed the role of FLT4 in the development of functional dysfunction in cerebrovascular accidents and created new opportunities for the study of this enzyme and its blockers in the formation of new therapeutic strategies.

## 1. Introduction

Ischemic stroke is one of the most common diseases in the world, characterized by high disability and death rates of patients and is especially significant in terms of life expectancy. In the coming decades, an increase in the average age of the population and additional risk factors such as hypertension, being overweight, and a sedentary lifestyle will contribute to the increase of the prevalence of pathologies related to the acute impairment of blood supply to the brain.

Ischemic damage triggers signaling pathways that lead to nerve cell death and destruction of the brain tissue. Oxygen and energy starvation activate pathogenetic reactions in various types of cells of the central nervous system (CNS), including neurons, astrocytes, microglia, and vascular endothelial cells. The most important of these reactions include excitotoxicity, mediated by the excessive release of excitatory neurotransmitters [1], as well as dysfunction of mitochondria and oxidative stress [2]. These processes lead to disturbances in the functional and metabolic activity of nerve cells. They ultimately trigger necrotic and apoptotic processes, which are associated with the loss of structural and functional elements of the neural network [3,4,5]. In addition, an important component of the pathophysiology of ischemic stroke is inflammation mediated by astrocytes and microglia [6,7,8,9].

Various kinases are components of almost all these molecular cascades; they can be considered potential molecular targets for the development of neuroprotection methods in ischemic stroke. For example, the neuroprotective role of phosphatidylinositol-3-kinase (PI3K)/Akt cascade has been widely studied [10,11,12]. PI3Ks are a family of proteins involved in intracellular signal transduction and the regulation of cell survival [13]. Previous investigations showed PI3K/Akt pathway plays a critical role to regulate cell activities, inflammatory response and apoptosis in cerebral ischemia. Akt, a serine protein kinase, triggers phosphorylation of several enzymes (e.g., glycogen synthase kinase 3 beta (GSK3β), thereby regulating a series of cellular functions [14,15]. However, the contribution of many neuronal kinases to the adaptation of nerve cells to ischemic damage and their effect on functional neural network activity has not yet been studied.

In our recent study [16], we performed screening of the effect of selective and nonselective blockers on more than 50 kinases with a previously undescribed neuroprotective effect on neuronal viability when modeling one of the factors of ischemic damage (glucose deprivation). Particular attention is paid to enzymes with neuroprotective effects that were not previously described as neuroprotectors or those kinases whose functions are not directly related to the function of cells of the nervous system. We identified several groups of kinases and characterized the most physiologically significant of them based on the effect they have. It was shown that inhibition of the eEF2K, SRC, and IKKb (IKK2) kinases maintain cell viability in primary neuronal cultures during in vitro glucose deprivation. In the present study, these kinases were selected to assess their effect on the neural network activity of brain cells in ischemic injury.

Unlike other tissues and organs, brain functionality depends not on individual cells, but rather on their functional ensembles, which make up neuron-glial networks. Neural networks are considered as the minimum functional unit that ensures the implementation of higher cognitive functions, including memory, consciousness, emotional reactions, and the ability to respond to environmental changes. To understand the pathogenesis and to develop methods of therapy for diseases of the central nervous system, it is essential to study the functional network activity of nerve cells.

The functional network activity is spontaneous spatiotemporal patterns of bioelectric signaling between nerve cells in neuronal assemblies. These rhythmic modulations in neuronal activity have been linked to various important brain functions, including attention, sensory or computational processing, decision-making and consciousness [17]. Calcium ions play a crucial role in signaling between nerve cells and the regulation of synaptic activity, and their imaging gives a means to estimate the functional state of neuronal networks with cellular resolution. The recently developed algorithm for analyzing calcium imaging data employs correlation analysis to reconstruct and follow the dynamical architecture of neuron-glial networks [18,19]. It provides with a powerful tool to characterize the collective and coordinated activity of these networks on the functional level and infer its response to model conditions, and evaluate the effect of damaging and protective factors. The goal of this work is to study the role of the four kinases belonging to different metabolic cascades in the adaptive potential of the neuron-glial network in modeling the key factors of ischemic damage.

## 2. Results

**Figure 1:**
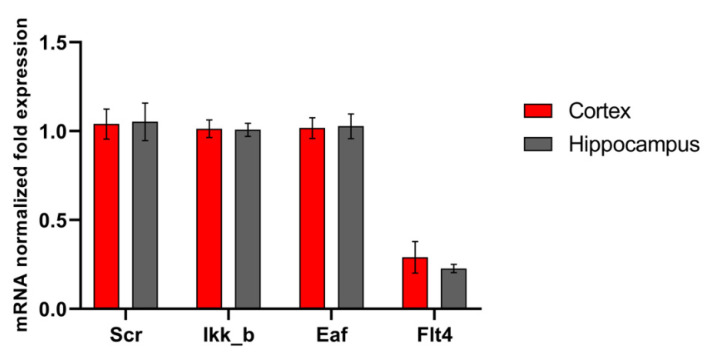
Features of kinase gene expression in primary cortical and hippocampal cultures. Data are normalized to the reference gene (Oaz1).

**Figure 2:**
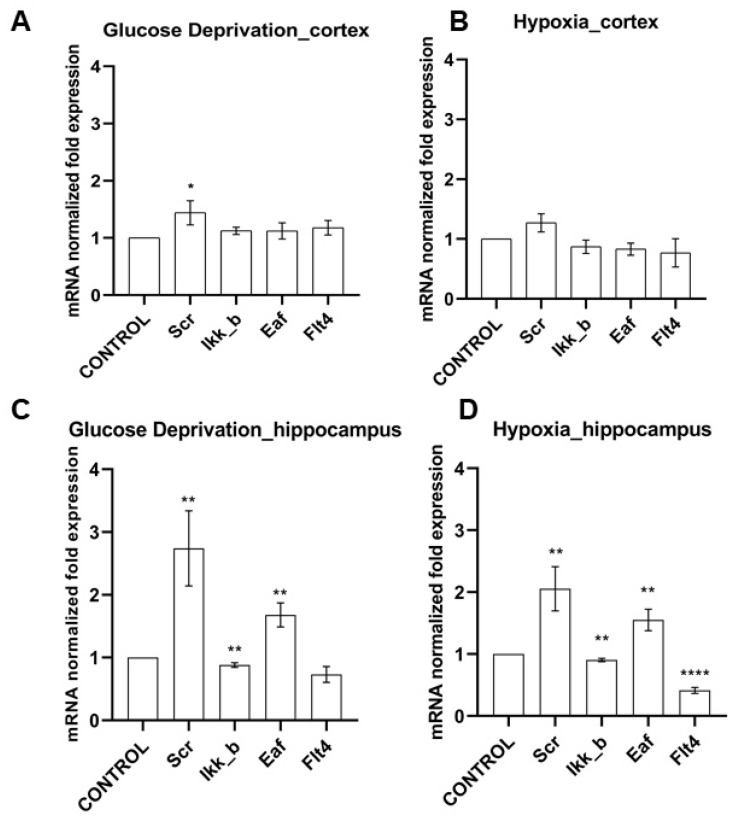
Features of kinase gene expression on day 7 of the post-hypoxic period. Data are normalized to the intact cultures (Control). * *p* < 0.05, ** *p* < 0.01, **** *p* < 0.001, the Mann-Whitney U test.

The functions of the studied kinases in the nervous system are so far almost undescribed. However, in our recent work, it was shown that their blockade has a positive effect on the viability of nerve cells in a deficiency of energy substrates, which indicates the important role of the selected kinases in the regulation of adaptation of nerve cells to stress-factors [16]. We carried out a study of the level of expression of the selected kinases by nerve cells of different parts of the brain under physiological conditions and during the modeling of damaging ischemic factors. Expression of all studied enzymes in the nervous tissue was demonstrated. No significant differences were found in the mRNA level of the corresponding kinases in primary hippocampal and cortical cultures (Figure 1). Next, we evaluated the influence of ischemic factors on the expression of kinases in these areas of the brain (Figure 2).

We have shown that the effect of ischemic factors on cells of the cerebral cortex does not significantly affect the level of mRNA expression of the studied kinases. When modeling hypoxia, the expression of the considered kinases did not change; when modeling glucose deprivation (GD), an increase in the expression of SRC kinase mRNA by 1.44 ± 0.36 times was noted.

It is interesting to note that the level of expression of all studied kinases in hippocampal cell cultures significantly changed when both ischemic factors were simulated (Figure 2). It was demonstrated that the expression of mRNA of kinases SRC and eEF2K increased significantly in the postischemic period (SRC when modeling GD by 2.74 ± 1.04 times, when modeling hypoxia by 2.06 ± 0.61; eEF2K by 1.68 ± 0.33 and 1.55 ± 0.3 times, respectively). The expression of IKKb kinase, on the contrary, decreased under the influence of both ischemic factors (with glucose deprivation by 13 ± 6.5%, with hypoxia by 9.4 ± 4.5%). Glucose deprivation did not affect the expression of FLT4 by hippocampal cells; hypoxia caused a significant decrease in the expression of this kinase by more than two times.

Thus, for further research, we chose hippocampal cell cultures as an object since they demonstrated a strong response to ischemic exposure by the kinome.

We evaluated the neuroprotective effect of the blockade of the studied kinases in the modeling of glucose deprivation and acute normobaric hypoxia in vitro in primary hippocampal cultures. It is important to note that, under normal conditions, the blockade of the selected kinases did not affect the viability of nerve cells, except for the blockade of the FLT4 kinase. The use of the inhibitor SAR-131675 under normal conditions led to a decrease in the viability of nerve cells (Table 1).

**Figure 3:**
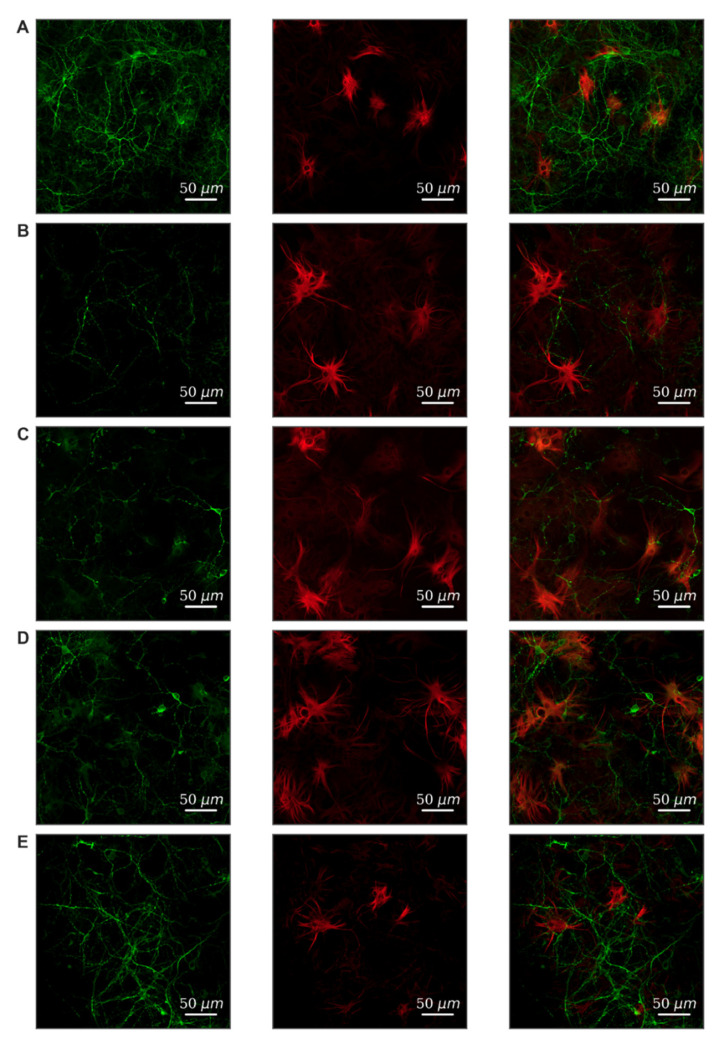
Morphology of primary hippocampal cultures on day 21 of cultivation in vitro. Green, stained by a marker of neuronal protein (MAP2); red, marker of cytoskeleton protein of differentiated astrocytes (GFAP). (**A**) Sham. (**B**) Hypoxia. (**C**) Glucose deprivation. (**D**) Hypoxia + FLT4 blocker. (**E**) Glucose deprivation + FLT4 blocker. Scale bars: 50 µm.

Modeling of both damaging factors led to a strong decrease in viability on the 7th day after exposure compared to the “Intact” group (Table 1). To verify the cellular content, primary hippocampal cultures were subjected to immunocytochemical staining. We have shown that neurons and astrocytes are present in primary cultures of the hippocampus in the 1:2 ratio (Figure 3A). Hypoxia leads to predominant neuronal death; the ratio of cell types changes and is 1:3.6 (Figure 3B). Fragmentation of neuronal processes should also be noted, which indicates the destruction of connections between cells and the loss of synapses. Glucose deprivation has a less pronounced effect on the morphology of cell cultures.

Inhibitors of SRC, Ikkb, and eEF2K kinases significantly increased the resistance of cells to the action of both key ischemic factors (Table 1). ACHP, an Ikkb kinase inhibitor, demonstrated the most significant neuroprotective effect. The blockade of FLT4 kinase with the SAR-131675 inhibitor had a neuroprotective effect only in the simulation of hypoxia.

We assessed the effect of blockade of the studied kinases on the calcium activity of primary hippocampal cultures under physiological conditions. It is important to note that, although the blockade of Ikkb and eEF2K kinases did not affect the viability of nerve cells, it modulated their calcium activity. The eEF2K blockade led to significant inhibition of calcium activity and a decrease in the number of cells in which calcium events were recorded from by 3.8 times (Table 2).

**Figure 4:**
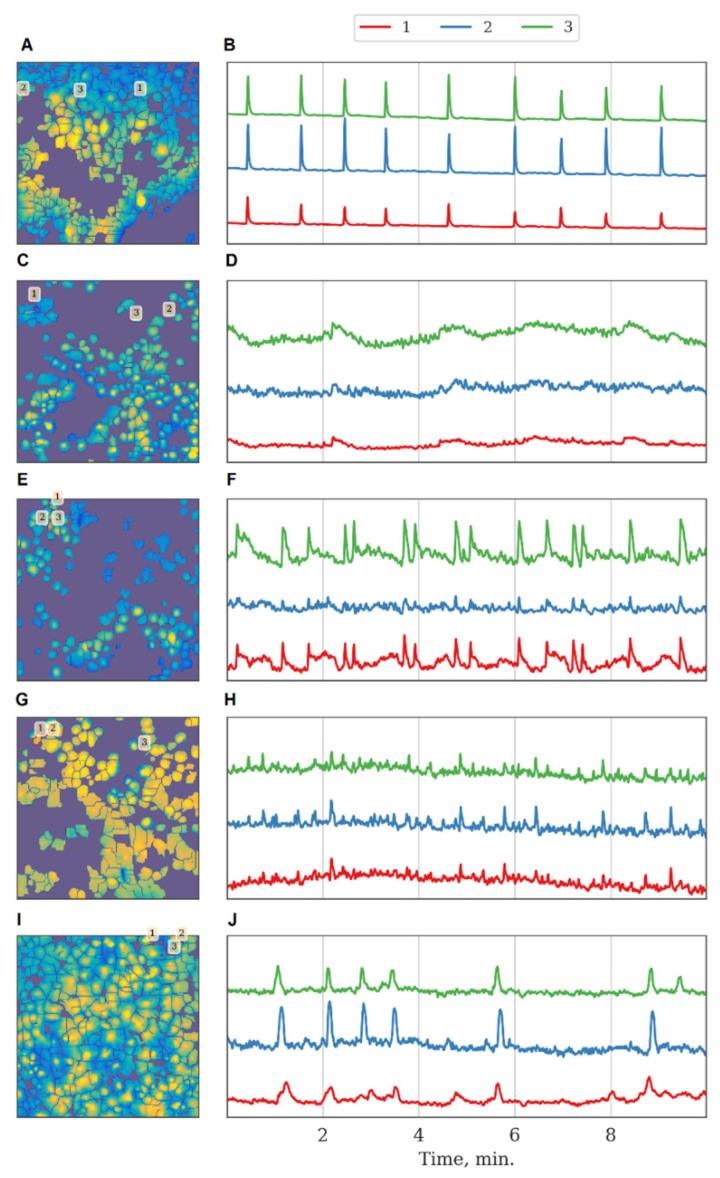
Characteristic examples of the dynamics of Oregon Green calcium sensor fluorescence in primary monoastrocytic cultures in vitro. (**A**,**B**) Sham, (**C**,**D**) eEF2k, (**E**,**F**) Ikkb, (**G**,**H**) FLT4, (**I**,**J**) SRC kinase.

The inhibition of Ikkb and FLT4 kinases led to a decrease in the duration of calcium oscillations. The SRC kinase was of particular interest because its blockade did not significantly affect the main characteristics of calcium activity under normal conditions. Representative examples of recordings of calcium activity together with kinase blockade are shown in Figure 4.

Next, we examined the calcium activity of primary hippocampal cultures when simulating ischemic factors together with the inhibition of kinases. It has been shown that both glucose deprivation and hypoxia led to inhibition of calcium activity. Hypoxia has a stronger negative impact on nerve cells. The number of cells with the recorded calcium events decreased by 23% when modeling GD, and by 42% when modeling hypoxia (Table 3). Furthermore, in the post-hypoxic period, there was a decrease in the incidence of calcium events.

The blockade of eEF2K kinase aggravated the negative effect of damaging factors on calcium dynamics in cells of primary hippocampal cultures. The number of active cells in the “GD + eEF2K” and “Hypoxia + eEF2K groups was significantly lower than in the “GD” and “Hypoxia”. The blockade of Ikkb kinase led to the aggravation of the disorders caused by glucose deprivation and had no effect on hypoxic damage.

The effects of SRC and FLT4 kinase blockers are of the greatest interest. With the blockade of SRC kinase, the number of cells exhibiting calcium activity in the presence of energy substrates deficiency did not differ from the intact group, however, under hypoxia, no protective effect was revealed for this parameter. The use of the SAR-131675 blocker, which inhibits the FLT4 kinase, maintained the number of cells in which calcium events were recorded under the influence of both hypoxia and glucose deprivation. However, in the “GD + FLT4” group, a decrease in the frequency and duration of calcium oscillations was noted. The number of active elements is fundamental for maintaining the functional activity of the neuron-glial network. Therefore, the effect of the FLT blocker can be considered protective.

We have studied the effect of FLT4 kinase blockade in modeling stress factors on primary hippocampal cultures’ cellular composition. The results are presented in Figure 3D,E. It can be noted that the blockade of FLT4 kinase contributes to the preservation of the morphology of neuronal processes and the maintenance of neuronal viability. This effect is expressed in the simulation of glucose deprivation and, to a lesser extent, in hypoxia simulation.

**Figure 5:**
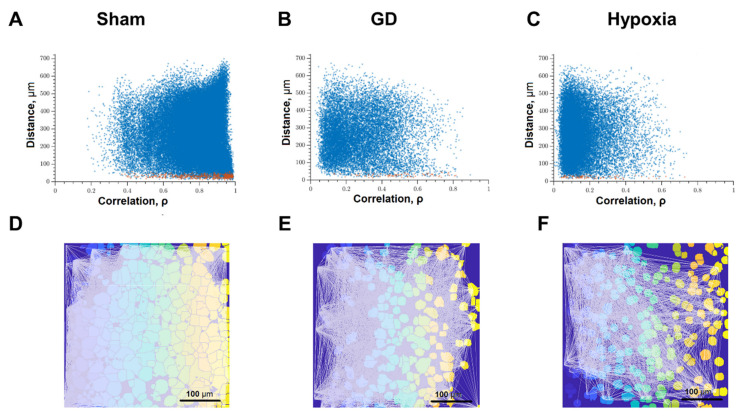
Correlation dependence between spontaneous Ca^2+^ oscillations and cell distance (red dots—pairs of neighboring cells, blue dots—pairs of distant cells): (**A**) Intact; (**B**) glucose deprivation, (**C**) hypoxia; representative correlation network graphs with a threshold of >0.3. (**D**) Intact; (**E**) glucose deprivation; (**F**) hypoxia.

The key aspect in the characterization of the dynamic interactions of neuron-glial networks in our study is the neural network parameters of calcium activity, which characterize the degree of connectivity of network elements after exposure to ischemic factors (Figure 5). Application of previously developed algorithms for network analysis of imaging data [19] makes it possible to comprehensively characterize the features of network activity reorganization under damaging influences.

Figure 5 analyzed the correlation between the level of correlation of calcium activity and the distance between pairs of cells in culture. Adjacent pairs of cells, the somas of which are in direct contact with each other, are shown as red dots; distant cells are shown as blue. Under normal conditions, both adjacent and distant cells have a high level of correlation; the signal correlation value is 0.50 [0.33–0.68] (Figure 5A). Thus, in the primary hippocampal cultures under physiological conditions, a developed dynamic neuron-glial network is formed, which is characterized by correlated calcium dynamics. The visualization of connections between functional elements of the network is shown in Figure 5D.

The impact of ischemic factors leads to a significant decrease in the connectivity of neural-glial networks and loss of functional connections (Figure 5B,C,E,F). The number of significant correlations decreases in both adjacent and distant pairs of cells (Figure 5B,C). For this parameter, the negative effect of oxygen deprivation on the connectivity of neural networks is stronger compared to the energy substrates deficiency. The correlation of calcium dynamics was 0.29 [0.16; 0.62] in the “GD” group and 0.22 [0.14; 0.45] in the “Hypoxia” group. It demonstrates a decrease in the connectivity of the neural-glial network and a loss of functional connections between nerve cells. The number of functionally significant cell connections in the “Sham” group was 391.67 [107.18; 574.29], “GD” 38.26 [1.66; 207.43], “Hypoxia” 147.47 [3.91; 329.22] (Figure 5C and Figure 7C).

**Figure 6:**
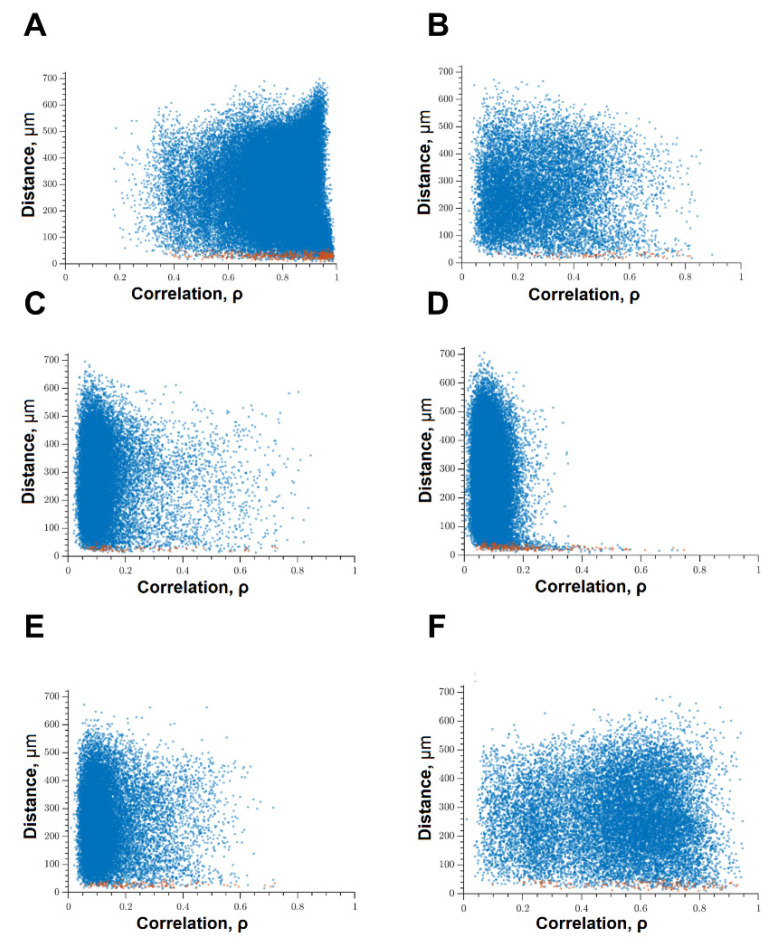
Correlation dependence between spontaneous Ca^2+^ oscillations and cell distance (red dots—pairs of neighboring cells, blue dots—pairs of distant cells): (**A**) Sham; (**B**) glucose deprivation, (**C**) GD + SRC, (**D**) GD + Ikkb, (**E**) GD + eEF2K, (**F**) GD + FLT4.

**Figure 7:**
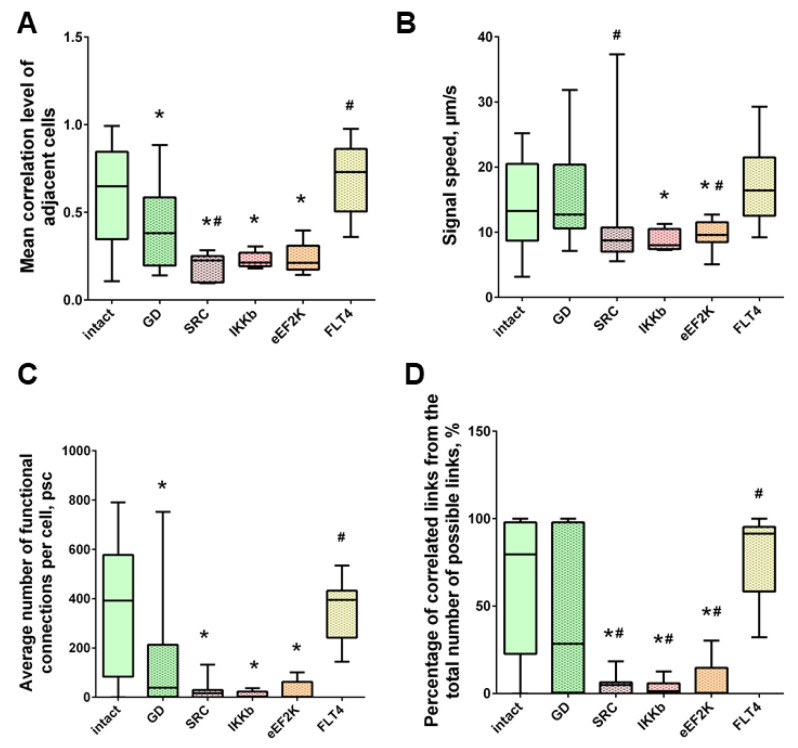
Neuron-glial network activity reorganization in primary hippocampal cultures. (**A**) Mean correlation level of adjacent cells; (**B**) the rate of signal delay between cells; (**C**) average number of functional connections per cell; (**D**) the percentage of correlated connections from the total number of possible connections. * versus “Intact”, # versus “GD”, *p* < 0.05, the Mann-Whitney test.

When modeling glucose deprivation of the studied kinases, only the blockade of FLT4 makes it possible to partially maintain the degree of correlation of the calcium activity of cells. The level of correlation of all cells does not differ from the indices of the intact group and is 0.56 [0.46; 0.61] (Figure 6). The level of activity correlation between pairs of both distant and adjacent cells in other experimental groups does not differ from the parameters of the “GD” group and is significantly lower than in the “Sham” group. Evaluation of other parameters of neural–glial network connectivity confirmed the neuroprotective effect of SAR-131675, a blocker of FLT4 kinase. Its use in modeling glucose deprivation makes it possible to maintain the number of functionally significant connections between cells, the rate of propagation of the calcium signal, and the degree of correlation between the activity of adjacent cells at the level of intact cultures (Figure 7).

**Figure 8:**
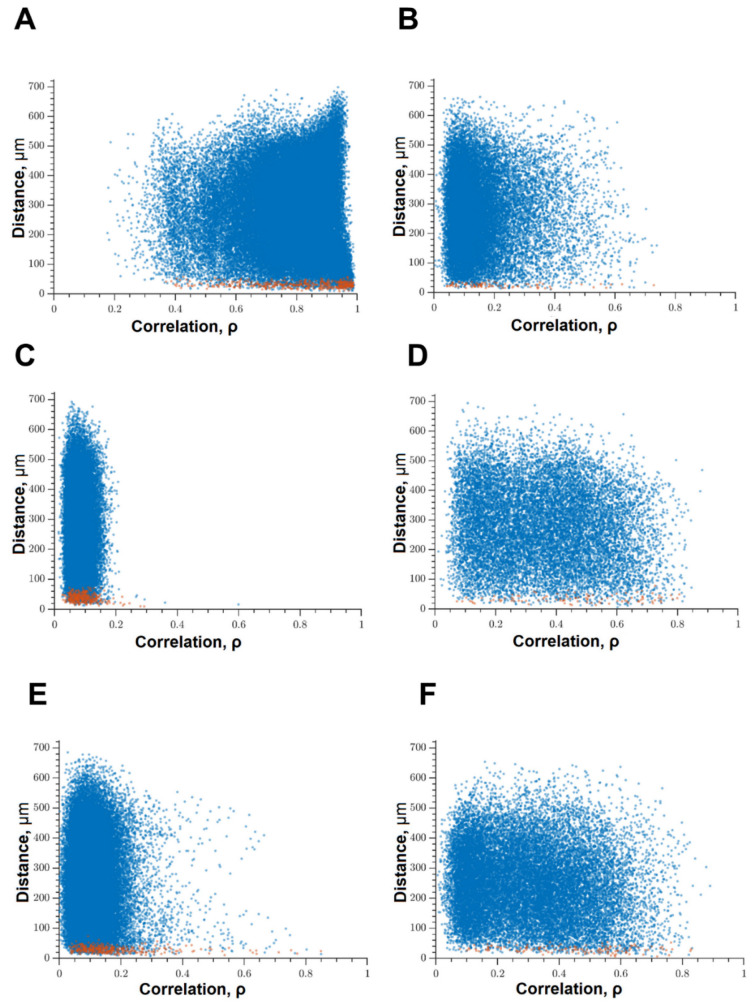
Correlation dependence between spontaneous Ca^2+^ oscillations and cell distance (red dots—pairs of neighboring cells, blue dots—pairs of distant cells): (**A**) Sham, (**B**) hypoxia, (**C**) hypoxia + SRC, (**D**) hypoxia + Ikkb, (**E**) hypoxia + eEF2K, (**F**) hypoxia + FLT4.

**Figure 9:**
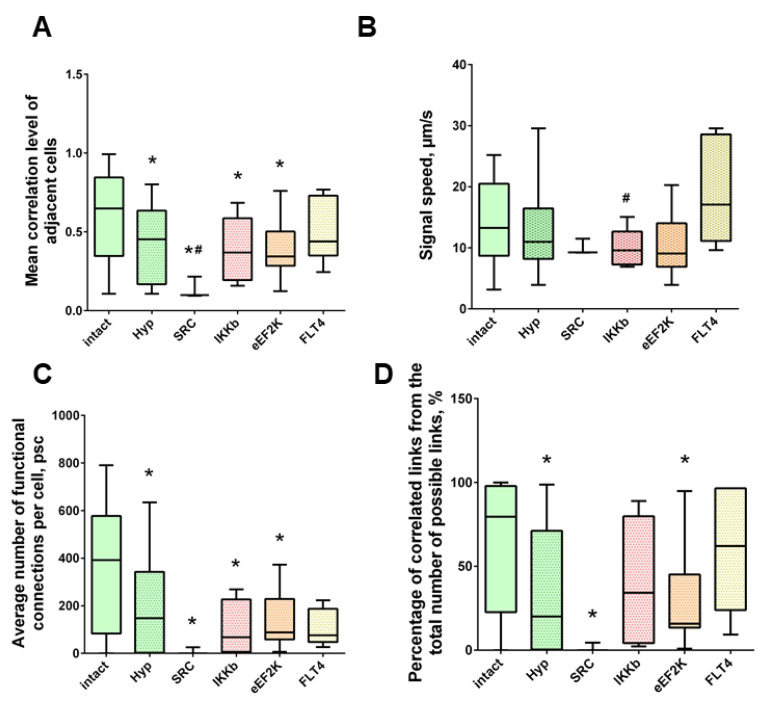
Neuron-glial network activity reorganization in primary hippocampal cultures. (**A**) Mean correlation level of adjacent cells; (**B**) the rate of signal delay between cells; (**C**) average number of functional connections per cell; (**D**) percentage of correlated connections from the total number of possible connections. * versus “Intact”, # versus “Hypoxia”, *p* < 0.05, Mann-Whitney test.

Under the influence of hypoxia, the destruction of network interactions is expressed more significantly in terms of the correlation of the activity of functional network elements. The most significant neuroprotective effect was shown in the blockade of FLT4 kinase in the simulation of hypoxia. The degree of correlation between adjacent pairs of cells and all cells in the culture, the rate of calcium signal propagation, and the proportion of functionally significant connections from their maximum possible number in the “Hypoxia + FLT4” group did not differ from the parameters of intact cultures (Figure 8 and Figure 9)**.**

Under hypoxic exposure, partial preservation of the network characteristics of calcium activity was observed in the “Hypoxia + Ikkb” group. The level of correlation of calcium signals was 0.33 [0.14; 0.47], which is significantly higher than in the “Hypoxia” group (0.22 [0.14; 0.45]). However, it should be noted that the correlation of activity between adjacent cells was significantly lower than in the intact group and did not differ from the “Hypoxia” group (Intact, 0.65 [0.40; 0.83]; Hypoxia, 0.45 [0.18; 0.63]; Hypoxia + Ikkb, 0.37 [0.21; 0.51]). Moreover, the percentage of correlated connections from the total number of possible connections did not differ from the indices of the intact group (Figure 9D).

## 3. Discussion

Our work was focused on identifying new targets for correcting the consequences of the impairment of cerebral circulation. To this end, we focused on studying the molecular and cellular mechanisms of action of kinome representatives with previously undescribed neuroprotective functions. When modeling individual ischemic factors, the blockade of all the kinases considered in the work preserves the viability of nerve cells effectively. However, in some cases, the functional activity of neuron-glial networks is not maintained, for example, in the blockade of eEF2K kinase. It is the preservation of a functionally complete interaction between the components of neuron-glial networks that is the basis for effective neuroprotection and is necessary to maintain the cognitive and amnestic functions of the brain. To assess the neuroprotective effect of blockade of various molecular targets, it is not enough to assess the survival of nerve cells. A key aspect for understanding the state of neuron-glial networks is the assessment of functional network activity. Calcium imaging is one of the relevant methods for recording the spatiotemporal patterns of neural network activity. Traditional electrophysiological methods make it possible to record either the activity of single neurons (patch-clamp) or small neuronal ensembles (multielectrode arrays), the individual cellular elements of which can be included in various functional networks. Their total activity does not allow making unambiguous conclusions about the functional network architecture. Visualization of calcium dynamics in the cytoplasm of nerve cells is an extremely informative approach for assessing neural network metabolic activity since it allows visualizing the architecture and mapping the activity of neural networks with cellular resolution since changes in the content of cytoplasmic transient-free calcium [Ca^2+^] are directly related to synaptic activity [20].

Among the kinase blockers studied, the most promising molecular targets for the development of methods for correcting and maintaining functional neural network activity are ACHP, which inhibits Ikkb kinases, and SAR-131675, which inhibits FLT4 kinase. On the other hand, the very fact of maintaining viability, without maintaining activity, could in the short term be considered as a favorable factor that allows cells to be preserved for possible stimulation of functional regeneration.

The choice of kinases as potential targets for the activation of nerve cells’ adaptive resources was determined by the results of screening of the effect of selective and nonselective blockers of more than 50 kinases, the effect of which on the viability of nerve cells in the modeling of ischemic factors in vitro has not been previously studied [16]. The four kinases, the blockade of which had the strongest neuroprotective effect, were studied in detail in this work.

The studied kinases belong to various metabolic pathways. The IκB kinase enzyme complex is part of the upstreaming NF-κB signal transduction. The effects of the activation of this nuclear transcription factor are multidirectional, which may be associated with the neuroprotective effect of Ikkb kinase blockade [21]. Our data demonstrate the neuroprotective effect of IkkB blockade—which is stronger than the hypoxia—and are consistent with the few available works indicating the potential neuroprotective properties of IKKb in cerebral ischemia. For example, the antioxidant pathway IKK/NF-κB/SOD2 is important for the protection of nerve cells during the development of excitotoxicity [22]. Experiments on mice with IKK2 deletion in astrocytes have shown that such animals are more resistant to nitrosative stress and demonstrate a decrease in the level of neuronal apoptosis when simulating Parkinson’s disease. This effect is assumed to be associated with inhibition of the astrocytic expression of inflammatory genes [23].

The SRC kinase was of great interest for us. We have shown that its inhibition makes it possible to maintain the viability of cells in primary cultures of the hippocampus when modeling the key factors of ischemia, significantly higher than it was in the control group. Kinases of the SRK family of nonreceptor kinases are involved in the regulation of many normal and pathological processes in the nervous system, including cell survival and proliferation, and angiogenesis. Although studies of the effect of SRC kinase on the resistance of the brain to ischemia have not been carried out, some works have shown the neuroprotective effect of inhibition of c-SRC kinase under the neurotoxic effects of kainic acid and oxidative stress [24]. The neuroprotective effect of blockade of SRC kinase may be explained by its ability to enhance the activity of NMDA receptors [25] which, under conditions of ischemic factors, can lead to the development of excitotoxicity. There is evidence that the use of inhibitors specific for Src effectively reduced hydrogen-peroxide-induced apoptosis of cells during oxidative stress [26]. Since oxidative stress develops during hypoxic damage, this may also be one of the mechanisms of the neuroprotective effect of SRC kinase blockade shown by us. However, our data indicate that, despite the effective maintenance of the viability of nerve cells, neural network activity is not preserved during the blockade of this kinase. Of particular interest are the data according to which the correlation characteristics of neural network activity are disturbed during glucose deprivation (despite the preservation of calcium events). Thus, nerve cells cease to interact with each other effectively.

The data on the role of FLT4 kinase in ischemic injury are of the greatest interest. There is little information available about its functions in the nervous system. FLT4 kinase (VEGFR3, vascular endothelial growth factor receptor 3) acts as a cell surface receptor for VEGFC and VEGFD (vascular endothelial growth factor) and promotes proliferation, survival, and migration of endothelial cells and regulates angiogenesis. Signaling with activated FLT4 results in enhanced production of VEGFC and to a lesser extent VEGFA, thereby creating a positive feedback loop that amplifies FLT4 signaling. It mediates the activation of the MAPK1/ERK2, MAPK3/ERK1 signaling pathway, the MAPK8 and JUN signaling pathway, and the AKT1 signaling pathway, which are involved in maintaining cell viability. It is known that VEGF has neurotrophic and neuroprotective activity in both the peripheral and central nervous systems, exerting a direct effect on neurons, Schwann cells, astrocytes, neural stem cells, and microglia. The involvement of FLT4 in the cascades initiating the maintenance of cell survival, neurogenesis, and angiogenesis, suggests that modulating the activity of VEGF receptors can be effective in the therapy of several neurodegenerative diseases and ischemic stroke. However, there are, so far, few data on this in the scientific literature. When simulating focal ischemia in rats, it was shown that blockade of FLT4 (VEGFR3) reduces activation of the lymphatic endothelium, reduces the level of activation of proinflammatory macrophages, and reduces the volume of cerebral infarction [27]. It has also been demonstrated that VEGFC and VEGFR-3 are involved in the neuroprotective effects of preconditioning in the mouse hippocampus.

FLT4 blockade can likely reduce the level of inflammation and reactivity of glial cells, since VEGFR-3 could be involved in the development of the inflammatory response of astrocytes in ischemic strokes [28,29]. At present, there is no doubt that astrocytes play an important role in modulating synaptic plasticity and neural network interactions [30], therefore, preventing the transition of astrocytes into the reactive state can positively affect neural network activity. An indirect confirmation of this may be the fact that there is evidence of increased expression of vascular endothelial growth factor and its receptors during neurodegenerative changes in Alzheimer’s disease. At the same time, a higher expression of VEGFB, FLT4, FLT1, and PGF in the prefrontal cortex was related to the stronger cognitive impairments in patients indicating impaired neural network function [31].

Our model made it possible to assess the neuroprotective effect of FLT4 blockade using the SAR-131675 inhibitor when modeling isolated ischemic factors. A significant result is the preservation of correlation characteristics and functional network architecture when using this blocker together with modeling ischemic injury. We were the first to describe the effect of FLT4 blockade on the functional calcium activity of nerve cells. Preservation of a functionally complete dynamic interaction between the components of neuron-glial networks allows us to consider FLT4 as a promising target for the development of methods of protection against cerebral ischemia and is of interest for future research.

The studies performed revealed the role of FLT4 in the development of functional dysfunction in cerebrovascular accidents and exposed new opportunities for the study of this enzyme and its blockers in the formation of new therapeutic strategies.

## 4. Materials and Methods

### 4.1. Ethics Statement

All experimental procedures were approved by the Bioethics Committee of Lobachevsky University and carried out in accordance with Act 708n (23 082010) of the Russian Federation National Ministry of Public Health, which states the rules of laboratory practice for the care and use of laboratory animals, and Council Directive 2010/63 EU of the European Parliament (22 September 2010) on the protection of animals used for scientific purposes. Pregnant C57BL/6 mice (day of gestation 18) were killed by cervical vertebra dislocation.

### 4.2. Isolation of Murine Primary Cortical and Hippocampal Cultures

Neuronal cells were obtained from mice embryos and cultured on coverslips (18 × 18 mm) pretreated with polyethyleneimine solution (1 mg/mL) (Merck KGaA, Darmstadt, Germany) according to the previously developed protocol described in [14]. Isolation of embryonic cortex or hippocampi was performed in Ca^2+^- and Mg^2+^-free phosphate-buffered saline (PBS) with subsequent enzymatic digestion with 0.25% trypsin-ethylenediaminetetraacetic acid (EDTA, Thermo Fisher, Waltham, MA, USA) for 20 min. After centrifugation (800 rpm for 3 min), the pellet of dissociated cells was seeded on coverslips at an approximate initial density of 7000–9000 cells/mm^2^. The primary neuronal cultures were grown in neurobasal medium (Thermo Fisher, Waltham, MA, USA), supplemented with 2% B27 (Thermo Fisher, Waltham, MA, USA), 0.5 mM L-glutamine (Invitrogen, 25030-024), and 0.4% fetal bovine serum (FBS; PanEco, Moscow, Russia) under constant conditions of 35.5 °C, 5% CO_2_, and a humidified atmosphere in a Binder C150 incubator (BINDER GmbH, Tuttlingen, Germany). A half-replenishment of the medium was performed once every three days [21].

### 4.3. Immunocytochemical Analysis

To verify the cellular content, primary hippocampal cultures were subjected to immunocytochemical staining on day 21 of culture development in vitro (DIV). The cultures were fixed with 4% paraformaldehyde for 15 min at room temperature, followed by incubation with a solution of 0.2% Triton X-100/PBS for effective cell permeabilization. For immunofluorescence reactions, the cultures were then incubated for 2 h in the presence of a polyclonal mouse anti-GFAP (glial fibrillary acidic protein, a marker of differentiated astrocytes) primary antibody (Sigma-Aldrich G3893, Merck KGaA, Darmstadt, Germany, 1:500 dilution) and polyclonal rabbit anti MAP2 (a marker of differentiated neurons) primary antibody (Abcam 32454, Cambridge, UK, 1:500 dilution). Next, the cultures were subjected to a 45-min incubation in the following secondary antibody mixture: goat anti-mouse Alexa 647 (Thermo Fisher Scientific, A21236, Waltham, MA, USA, 1:800 dilution) and chicken anti-Rabbit Alexa Fluor 488 (Thermo Fisher Scientific, A21441, Waltham, MA, USA, 1:800 dilution). The stained material was observed using a Zeiss LSM 800 fluorescence confocal microscope (Carl Zeiss, Oberkochen, Germany).

### 4.4. Glucose Deprivation

Modeling of glucose deprivation in vitro was performed on the 14th day of cultivation (DIV) of primary cultures by replacing the normal culture medium with a medium that did not contain glucose, lactate, and pyruvate (PanEco, Moscow, Russia) for one hour, followed by a reverse replacement of the medium with a standard one.

### 4.5. Acute Normobaric Hypoxia Model

Acute normobaric hypoxia was modeled on day 14 of culture development in vitro (DIV) by replacing the normoxic culture medium with a medium containing low oxygen for 10 min. The oxygen was displaced from the medium in a sealed chamber with an inert gas (argon). The oxygen concentration in the culture medium was decreased from 3.26 mL/L to 0.37 mL/L [21]. After 10 min of incubation, the hypoxic medium was replaced by a complete culture medium.

### 4.6. Real-Time PCR

Total RNA was extracted from primary dissociated neuronal cultures of the mouse cerebral cortex and hippocampus using the ExtractRNA reagent (Evrogen, Moscow, Russia); reverse transcription was performed using the MMLV RT kit and the random primer mixture (Evrogen, Moscow, Russia). RT-qPCR reactions were performed using the qPCRmix-HS SYBG Green kit (Evrogen, Moscow, Russia) using an Applied Biosystems 7500 RT-PCR amplifier. The reaction was set up in four repetitions using the following cycle protocol: 50 °C for two minutes, 95 °C for 10 min, followed by 40 cycles at 95 °C for 15 s and 62 °C for one minute, then 95 °C for 15 s, 60 °C for one minute, and 95 °C for 30 s. The primers used are shown in Table 1. The sequence of the cycles for the various samples is shown in Table 4.

Data processing was carried out using the ΔΔC method and a control sample, in which the level of the target gene was taken as a unit. The *Oaz1* gene was used as a housekeeping gene to normalize the findings.

### 4.7. Pharmacological Treatment

Application of kinase inhibitors was carried out on the 14th day of cultivation, 20 min before the modeling of the ischemia factor, and immediately during the modeling at a concentration of 1 μM. The chemical structure of the inhibitors is shown in Table 5. The kinase inhibitors used were kindly provided the Drug Discovery Group, Ontario Institute for Cancer Research, Toronto, ON, Canada.

### 4.8. Cell Viability Assay

The viability of primary hippocampal cultures was expressed as a ratio of the number of nuclei of dead cells stained with propidium iodide (Merck KGaA, Darmstadt, Germany) to the total number of cells stained with bis-benzimide (Merck KGaA, Darmstadt, Germany). Propidium iodide and bis-benzimide at concentrations of 5 μg/mL and 1 μg/mL, respectively, were added to the culture 30 min before registration. The stained cultures were observed using a ZEISS Observer A1 inverted fluorescence microscope (Carl Zeiss, Oberkochen, Germany) with a 20×/0.2 objective.

### 4.9. Calcium Imaging

To characterize the dynamics of calcium homeostasis in the hippocampal cells, functional calcium imaging was performed with a fluorescent calcium-sensitive dye Oregon Green 488 BAPTA-1 AM (OGB-1) (Invitrogen, O-6807, Carlsbad, CA, USA) on an LSM 510 confocal laser scanning microscope (Carl Zeiss, Oberkochen, Germany) with a W Plan-Apochromat 20 ×/1.0 objective. The calcium sensor, 0.4 μM OGB-1 (Thermo Fisher Scientific, Waltham, MA, USA), was dissolved in DMSO (Merck KGaA, Darmstadt, Germany) with 4% pluronic F-127 (Thermo Fisher Scientific, Waltham, MA, USA), added to the examined cultures and incubated for 30 min in a CO_2_-incubator. The OGB1 fluorescence excitation wavelength was set at 488 nm with an argon laser, and the emission was recorded using a 500–530 nm filter. To evaluate the dynamics of changes in the intracellular calcium concentration, a time-series of confocal images was recorded. The registration rate used was two frames per second. The spontaneous calcium activity of primary hippocampal cultures was recorded on day 7 post-ischemic damage. The following main parameters were analyzed: duration of calcium events (time period from the beginning to the end of an oscillation, s), frequency of calcium oscillations (average number of oscillations per min), and percentage of active cells (the cells number with at least one recorded oscillation divided by the total cell number, %) [18,21].

### 4.10. Calcium Network Activity Analysis

Calcium network analysis was performed using a previously developed algorithm [19]. The approach to the reconstruction of the network consisted of two steps: computation of pairwise correlations of the preprocessed fluctuations in the intracellular calcium level of individual cells (network nodes) and applying a threshold cutoff for the correlation level ρ > 0.3 to determine the significant network connections. The threshold correlation level corresponds to the upper bound of the correlation of a primary monoastrocyte culture in the absence of neurons. Exceeding the threshold of the correlation in calcium activity above 0.3 indicates significant network activity of neurons.

In the paper by [19], several measures were proposed to estimate network activity: the average level of correlation between adjacent cells, the average number of functional connections per cell, the percentage of correlated connections, and the average propagation speed of delays. The average level of correlation between adjacent cells shows how the strength of physical connections required for network communication. An average number of functional connections per cell describes the average number of cells whose calcium activity significantly correlates with the individual cell in the network. The percentage of correlated connections from the total number of possible connections quantifies the used resource of significant correlations in the network. A 0% value means the complete absence of significant correlative interactions. A 100% value characterizes the maximally possible level of significant correlations in the network. An increase in the last two parameters considered above indicates the presence of a long-term significantly correlated response of the cell network, whereas their low levels characterize its absence [18,19].

The propagation speed of calcium waves in the cell culture is associated with spatiotemporal delays. To estimate propagation speed, the average propagation speed of delays was introduced based on two parameters: the distance between corresponding somas and cross-correlation time delay between fluctuations in the calcium level of cell pairs. The average ratio of these quantities shows how far the wave peaks travel per unit of time.

### 4.11. Statistical Analysis

Quantitative results are presented as a mean ± standard mean error (SEM) for normal distributions, or as a median value and second and third interquartile range. To compare the two unrelated groups, the Mann-Whitney U test and ANOVA was used. The Tukey post hoc test was used as a post hoc test following ANOVA. At least three independent biological replicates were used for all experiments. The difference between the groups was considered significant if the p-value was less than 0.05.

## 5. Conclusions

The studies performed revealed the role of FLT4 in the development of functional dysfunction in cerebrovascular accidents and exposed new opportunities for the study of this enzyme and its blockers in the formation of new therapeutic strategies.

## Figures and Tables

**Table 1:** Cell viability of primary hippocampal cultures on day 7 post-hypoxia or post-GD modeling with kinase blockers application.

Group	Normal Condition Number of Viable Cells, %	GD Number of Viable Cells, %	Hypoxia Number of Viable Cells, %
Sham	96.05 ± 2.29		
Ischemic factor		78.79 ± 1.92 *	75.77 ± 2.17 *
SRC	92.61 ± 0.69	88.33 ± 1.21 *^,#^	81.64 ± 2.32 *^,#^
Ikkb	89.4 ± 1.49	90.32 ± 1.2 *^,#^	94.83 ± 1.39 ^#^
eEF2K	92.53 ± 1.12	89.705 ± 1.76 *^,#^	81.12 ± 1.86 *^,#^
FLT4	82.29 ± 1.9 *	76.98 ± 1.98 *	82.75 ± 1.61 *^,#^

*—versus “Sham”, ^#^—versus “Hypoxia” or “Glucose deprivation”, *p* < 0.05, two-way ANOVA and Tukey post hoc test.

**Table 2:** Main parameters of calcium activity of primary hippocampal cultures together with the blockade of intracellular kinases.

Group	Active Cells, % ^1^	Frequency, osc/min	Duration, s
Sham	61.13 ± 4.21	1.43 ± 0.27	11.79 ± 0.88
SRC	69.34 ± 9.46	1.11 ± 0.13	12.41 ± 0.3
Ikkb	65.95 ± 3.524	1.54 ± 0.42	7.75 ± 0.88 *
eEF2K	15.96 ± 2.09 *	0.54 ± 0.12 *	12.88 ± 0.67
FLT4	57.06 ± 28.07	2.13 ± 0.53	7.002 ± 1.05 *

^1^ Active cells, %—the cell number with at least one recorded oscillation divided by the total cell number; * versus “Sham”, *p* < 0.05, two-way ANOVA and Tukey post hoc test.

**Table 3:** The main parameters of the calcium activity of primary hippocampal cultures under the influence of ischemic factors together with the blockade of intracellular kinases.

Group	Active Cells, % ^1^	Frequency, osc/min	Duration, s
Sham	61.13 ± 4.21	1.43 ± 0.27	11.79 ± 0.88
GD control	47.17 ± 7.05 *	1.04 ± 0.25	11.77 ± 0.67
Hypoxia control	35.91 ± 1.05 *	0.48 ± 0.12 *	11.95 ± 0.36
GD + SRC	51.97 ± 5.79	1.03 ± 0.41	11.44 ± 0.705
Hypoxia + SRC	35.87 ± 5.67 *	1.03 ± 0.41	11.35 ± 0.07 ^#^
GD + Ikkb	21.54 ± 4.58 *^,#^	0.62 ± 0.11 *^,#^	15.93 ± 1.69 *^,#^
Hypoxia + Ikkb	38.69 ± 6.58 *	1.17 ± 0.21 ^@^	11.08 ± 1.03
GD + eEF2K	18.41 ± 2.55 *^,#^	0.81 ± 0.13 *	17.67 ± 2.68 *^,#^
Hypoxia + eEF2K	10.67 ± 2.05 *^,@^	1.15 ± 0.29 ^@^	9.38 ± 0.62 ^@^
GD + FLT4 (VEGFR3)	67.74 ± 9.33 ^#^	0.58 ± 0.08 *^,#^	8.94 ± 0.79 *^,#^
Hypoxia + FLT4 (VEGFR3)	63.58 ± 5.15 ^@^	0.87 ± 0.11 ^@^	11.66 ± 1.83

^1^ Active cells, %—the cell number with at least one recorded oscillation divided by the total cell number; * versus “Sham”, *p* < 0.05; ^#^ versus “Control GD”, *p* < 0.05; ^@^ versus “Control Hypoxia”, *p* < 0.05, two-way ANOVA and Tukey post hoc test.

**Table 4:** The primer sequences used for RT-qPCR target the following genes.

Gene	Forward Primer	Reverse Primer
Ikkb	5′-AACCAGAATCCAGGAAGACACG-3′	5′-TCGTTTGTCTTGCTGTCTGAGATG-3′
Flt4	5′-AACAACACGGGCAGCTACCACT-3′	5′-CAGGAGCGTGTCAGGTTTGTTGA-3′
Scr (SRC)	5′-AGGCTTCAACTCCTCGGACA-3′	5′-TTCTTGAAGGACAGGTCAGTCTCT-3′
Eaf (eEF2K)	5′-TCGAGGACATCGCCACAGA-3′	5′-GTTTCTTCGTCCTGAAGCACTC-3′
Oaz1	5′-AAGGACAGTTTTGCAGCTCTCC-3′	5′-TCTGTCCTCACGGTTCTTGGG-3′

**Table 5:** The chemical structure of the kinase inhibitors.

Group	Kinase Blockers	Structural Formula
Intact	-	
Ikkb	ACHP	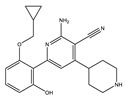
		C_21_H_24_N_4_O_2_
eEF2K	Compound 34	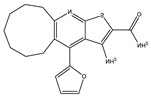
		C_19_H_21_N_3_O_2_S
SRC	Compound 4	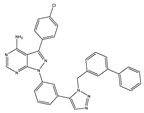
		C_32_H_23_ClN_8_
FLT4 (VEGFR3)	SAR-131675	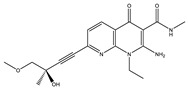
		C_18_H_22_N_4_O_4_

## Data Availability

The data used to support the findings of this study are available from the corresponding author upon request.
